# Determining Factors in the Use of Urban Parks That Influence the Practice of Physical Activity in Children: A Systematic Review

**DOI:** 10.3390/ijerph18073648

**Published:** 2021-03-31

**Authors:** Rosario Padial-Ruz, Mª Esther Puga-González, Álvaro Céspedes-Jiménez, David Cabello-Manrique

**Affiliations:** 1 Department of Didactics of Musical, Plastic and Corporal, University of Granada, 18011 Granada, Spain; rpadial@ugr.es; 2 Department of Physical Education and Sports, University of Granada, 18011 Granada, Spain; alvarocespedesjimenez@gmail.com (Á.C.-J.); dcabello@ugr.es (D.C.-M.)

**Keywords:** urban parks, children, user perception, design, physical activity, health

## Abstract

The design and/or remodelling of urban parks is a good health strategy to alleviate the lack of physical activity (PA) in children and, consequently, the different health problems derived from this. The main objective of the present study was to obtain a systematic review of the design features and characteristics that influence users’ visits to urban parks and the PA engagement in them. A literature search was carried out in the Web of Science (WOS) and Scopus databases during the months of June and July 2020. After considering and applying inclusion criteria, the final review sample was formed of 31 scientific papers published between 2010–2020. The results obtained in the review lead us to conclude that the needs of the population (children and family members who care for them) and socio-economic context of the area in which they are built must be considered when constructing and/or remodelling parks. Involving community members in playground renovations can have a positive effect on park use and PA engagement in children.

## 1. Introduction

The high rates of inactivity and sedentariness amongst children worldwide is a major problem that needs to be addressed immediately [1]. World Health Organisation (WHO) recommendations for physical activity (PA) in young people, aged 5–17 years, state at least 60 min per day of moderate to vigorous intensity activity. This activity should be mostly aerobic and should incorporate vigorous activities that particularly strengthen muscles and bones at least three times per week. However, over the past few years, we have seen an increase in the proportion of the population that does not comply with these recommendations. This includes a large number of children [2,3], with more children also coming from disadvantaged settings [4].

Inactivity at early ages can lead to high obesity rates. Excess body fat in the early stages of life can have serious health consequences at later stages, since the risk of contracting diseases derived from inactivity, such as diabetes, hypertension and coronary diseases, among others, is high and dangerous [5]. Thus, according to Omorou et al. [6], lack of PA may increase the likelihood of being obese in adulthood.

Another consequence of childhood obesity comes from the association between motor skills, PA and body composition in the pre-school stage. Diminished interaction with natural environments and the restriction of movement at increasingly younger ages can lead to poor execution of the motor tasks necessary for correct psychomotor development [7].

At a motor level, children will also present greater difficulties at a later stage when doing sports. Children with higher physical activity levels and less sedentary behaviour present better motor skills compared with those who mostly engage in sedentary pursuits, with the latter often unable to develop such skills adequately [8]. Nonetheless, inactivity not only causes problems at a physical and physiological level, but it also plays a fundamental role with regards to other areas, such as social and psychological areas. In the case of the psychological area, there is a greater probability of suffering from poor social and psychological health, bringing into play self-esteem issues, poor self-concept and other more serious problems such as depression and social discrimination [9,10].

The acquisition of a healthy lifestyle is a global need. For the population to have appropriate habits, it is important to intervene at crucial stages such as childhood and adolescence. The family, school and peers are responsible for encouraging these patterns in children as they are the ones who shape their daily environment [3,11]. Such intervention will contribute to favourable development at later stages, such as adulthood and old age, directly improving the quality of life of all individuals [12]. Sedentary behaviour experienced during childhood may become characteristic in individuals at latter stages. This can generate problems at future stages. It is, therefore, of vital importance to promote behaviours that succeed in motivating children to perform physical exercise [13]. Several authors have defended the need to incorporate healthy lifestyle habits during the first years of children’s lives [14,15]. In this way, Poeta et al. [14] urge the need to perform physical exercise from two to six years old, as weight is put on more quickly at this stage. Further, Haines et al. [15] argue that the acquisition of physical activity habits and adequate nutrition will protect children from noncommunicable diseases and obesity.

Furthermore, the regular practice of PA in school-age children, at adequate levels, is associated with important short- and long-term health benefits. It is considered protective against diseases, it improves aspects related to mental and emotional health, it improves academic performance [3,16,17,18] and it can even become a form of cultural and social transformation [19]. In addition, there is a reciprocal relationship between physical activity engagement at these ages and development of the motor skills and physical competencies that are fundamental for health [20,21]. Existing studies at early ages, such as those conducted by Carson et al. [22], Stanley et al. [23] and Timmons et al. [24], have identified positive associations between PA and adiposity, bone and skeletal, cardiometabolic, psychosocial and cognitive health [25,26], and motor skill development.

Schools are ideal environments for PA promotion to students. They offer a number of opportunities, including breaktime and physical education classes, etc. Classrooms represent ideal spaces for PA, although they are not always used for such purposes. This suggests the potential for achieving PA at different times and in different spaces (physical or motor education classes, active breaks, active pauses, physical education (PE) integrated within the curriculum, etc.), with this possibly having a very positive effect on behaviour [27], cognitive function and academic performance [26]. However, the situation we are experiencing today due to Covid19 has led to a drastic reduction in PA and concomitant increase in sedentarism, bringing with it serious consequences. Thus, now more than ever, a rethinking of healthy habits in the population is required, particularly in children. This must combine PA at home with outdoor activities and sports [28,29]. It is very important to provide children with opportunities to exercise, and it is necessary to have safe, accessible and outdoor recreational spaces [30].

Time spent in the open air is related to positive health outcomes. For instance, it reduces the risk of immune-based diseases [31], childhood stress, symptoms of attention deficit disorders, depression, asthma, etc. and generates a sense of well-being in the child [30]. In addition to physical health, mental health benefits in children have been improved following contact with the natural environment. Activities such as exploration encourage learning, attention and reflective practice [32], development of spirituality [33] and environmental awareness, which will lead to sustainable lifestyles [34].

To achieve health-related behavioural habits within the population, socioecological models state that certain healthy behaviours are influenced by the individual, social and physical characteristics of the environment [35,36,37,38,39,40,41], with these having the potential to contribute positively to health promotion. Existing research based on these theories focuses on the influence that physical characteristics and the construction of the environment have on health behaviour and visitation frequency, in addition to the PA and social interaction that takes place in parks and green spaces [42,43,44].

Within the built environment, parks and urban green spaces are related to PA. They provide a fundamental framework for alleviating sedentary lifestyles amongst children and the rest of the population in general. Most offer open spaces which are accessible to all at no or little cost [45]. They are important settings for children to develop their motor skills, physical capacity and cognitive and social abilities [46]. The design of these spaces to promote outdoor PA is, therefore, an effective and easy way of instilling active lifestyles in children, regardless of age and social status [47]. Increasing the frequency of visits to parks will promote increased PA [48].

Although most urban neighbourhoods in developed countries have parks and green areas, these are rarely visited by the population [49,50]. This is often because of the social environment surrounding them (insecure environments) [51] and sometimes because of their unattractive design [52,53]. Currently, apart from the exceptionality of the situation experienced by Covid19, parental concern about road safety and the danger posed by strangers is one of the reasons why the number of children going to parks has reduced. This has led to a limiting of children’s independent mobility and their resultant PA engagement [53]. Another motive for park avoidance is its design. Authors such as Hyndman et al. [54] suggest that it is important that the characteristics and design of the park are appropriate because not all parks are the same in terms of their potential for promoting PA [55]. Factors such as the amount of space available, layout and safety of the park should be carefully considered when designing a playground project. Most playgrounds comprise a “traditional” design (understanding by traditional parks such as those provided with metal structures such as swings, slides, seesaws, ladders and fireman’s sticks.). This may be one of the reasons why the number of visitors to such places is decreasing. Therefore, planners should take into account the factors that attract different groups of children to parks [50], as well as better understanding of how parks can be designed or redesigned in order to ensure optimal use by children and encourage increased PA from an early age [56,57].

The aim of the present study was to carry out a systematic review of the scientific literature in order to obtain a deep insight into the factors that can influence the use of parks and the increase of PA in them, both from the point of view of design and perception of users of playgrounds.

## 2. Materials and Methods

The present study follows the guidelines of the PRISMA statement (Preferred Reporting Items for Systematic reviews and Meta-Analyses) [58,59].

### 2.1. Eligibility Criteria

After establishing the study population, the research sample was selected according to the following inclusion criteria: (1) scientific papers focused on the search terms; (2) published between 2010–2020; (3) focused on the user and infrastructure characteristics of urban park design for public use; (4) interventions aimed at improving physical activity in children aged 1–12 years; (5) perceptions of parents or caregivers on the level of satisfaction of the parks. The age range selected in the inclusion criteria (1 to 12 years) is justified given the importance of the acquisition of health habits at these ages, being also a stage where the level of sedentary lifestyle has increased considerably in recent years. Papers were also included whose sample contained, in addition to the selected age, other age ranges since they were multigenerational studies. Those papers that focused exclusively on adolescents and those that did not seek to improve the quantity and quality of physical activity of the ages under study were discarded.

### 2.2. Information Sources and Search Strategies

The literature search was conducted during June and July 2020, using Web of Science (WOS) and Scopus databases as the main search engines. For initial article selection, references were analysed which examined the characteristics and designs of public urban parks whilst also considering users’ perceptions of them, with the aim of increasing their use and physical activity engagement in children. To this end, we used the terms “playgrounds”, “urban parks”, “physical activity” and “children” as keywords, using “and” and “or” as Boolean operators. We limited the time range of publication to the last decade (2010–2020) and only considered references published in English and Spanish. All scientific papers were included without excluding any research design. In this way, a total of 235 publications were obtained. Once the population was selected, (1) scientific papers focused on search terms were applied as the first inclusion criterion. This criterion was applied by first reading the title and summary of the study population to check that the paper met the first criterion. Subsequently, a systematic reading of the full text was carried out in order to apply the remaining criteria: (2) published between 2010–2020; (3) focused on the user and infrastructure characteristics of urban park design for public use; (4) interventions aimed at improving physical activity in children aged 1–12 years; (5) perceptions of parents or caregivers on the level of satisfaction of the parks.

**Figure 1:**
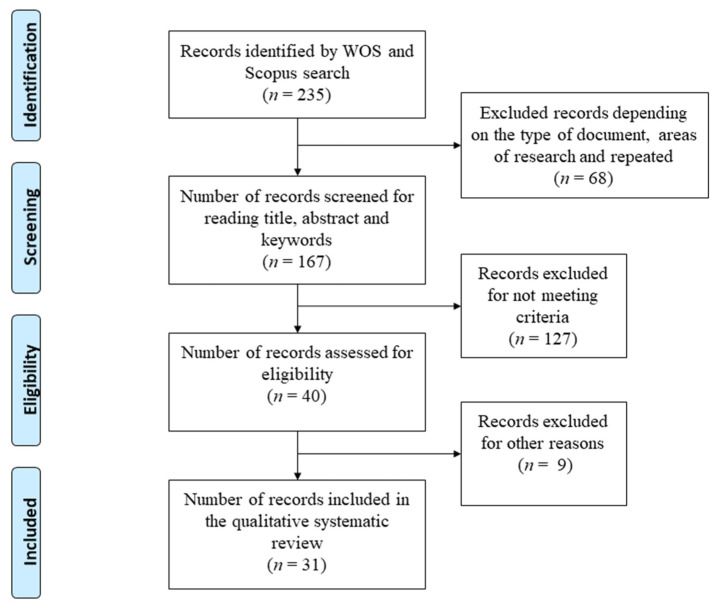
Flowchart of research paper selection.

Those papers that focused exclusively on adolescents and those that did not seek to improve the quantity and quality of physical activity of the ages under study were discarded. Finally, selected papers were organised and assigned an identification number (Figure 1).

### 2.3. Data Collection Process

To organise the findings of eligible papers, a data extraction form was developed and tested on a sample of included studies (*n* = 31). Data collection was done by the first author and reviewed again by the second author. For any discrepancies, the authors had a discussion to reach a consensus. Two tables (Table 1 and Table 2) were created by recording and coding the following data for each eligible paper: Table 1: (1) paper; (2) authors; (3) year of the studies; (4) country; (5) age of children and adults observed and interviewed; (6) sample; (7) park type; (8) context. Table 2 presents data for (1) study objective; (2) study type; (3) variables analysed in the study; (4) data collection instruments; (5) conclusion.

### 2.4. Risk of Bias in Studies

This study was coded by two of the authors in order to verify the reliability of the coding and the degree of agreement between the investigators for the selection and extraction of the data [84]. The risk of bias of each eligible article was assessed by adopting a dichotomous nominal scale of two unique values (yes/no), which was developed to assess concordance in the 31 studies in the sample. As variables of the scale, the inclusion and exclusion criteria indicated in Section 2.1 were taken (eligibility criteria). The degree of agreement obtained in the classification of the papers was 93%, which was obtained by dividing the number of coincidences by the total number of categories defined for each study and multiplying it by 100.

## 3. Results

### 3.1. Study Selection

As shown in Figure 1, a total of 235 papers were identified from the electronic databases WOS and SCOPUS, 195 of which were discarded after the evaluation of the abstracts, and the eligibility of the full texts of the 40 papers remaining was examined. Finally, 31 papers were selected and extracted, in which a total of 131,607 children (0 to 17 years) and 3526 adults (18 years and older) participated, and 1046 parks had been analysed.

### 3.2. Characteristics of Eligible Studies

#### 3.2.1. Outcomes Pertaining to Park Type

Following analysis of the articles obtained, it can be seen that 32.5% (*n* = 10) of studied parks (Table 3) corresponded to traditional urban parks; 35.48% (*n* = 11) were focused on children’s parks (playground) and the remaining 32.25% (*n* = 10) consisted of remodelled, innovative, contemporary and sustainable parks with special characteristics.

#### 3.2.2. Outcomes Pertaining to the Socio-Economic Context

Based on the classification on the economic context found in most of the papers in the sample, three categories were distinguished: high, medium and low socioeconomic levels. In those studies in which the socioeconomic level of the park’s users was not specifically specified, data such as the poverty index, GDP and unemployment rate of the city, region or state where the parks were located were reviewed.

As can be seen in Table 4, almost 39% of included studies were carried out in medium-low socio-economic contexts, whilst around 30% focused on plural contexts which covered all socio-economic categories. Only 19.35% of considered research was directed towards upper-middle class users.

#### 3.2.3. Outcomes Pertaining to Analysed Variables

The most commonly considered variables in the 31 studies (Table 5) corresponded to the “park characteristics” group, with the variable “characteristics of the park”/“equipment”/“design” being particularly prevalent, appearing in 14 papers (45.16%). Variables referring to PA were also considered by a large number of papers, for example the variables “physical activity intensity” and “physical activity levels” which accounted for 54% of the total (*n* = 17).

Within the group relating to park use, the variables “frequency”, “number” or “density” should also be mentioned, amounting to 48.38% (*n* = 15) of all considered variables.

With regards to park users, inclusion of the variables “age” and “sex” on 13 occasions was notable, as was the inclusion of “ethnicity” by seven papers (all in the USA).

#### 3.2.4. Outcomes Pertaining to Employed Instruments

Among the instruments used, we found that 70% of the 31 papers analysed used questionnaires, surveys or interviews. In addition, more than half (51.61%) used observation techniques, the most important of which was the System for Observing Play and Recreation in Communities (SOPARC) (12 papers). Almost 30% of the research studies utilised some type of instrument related to the location and density of parks. Only one study carried out a physical fitness test (Table 6).

#### 3.2.5. Outcomes Pertaining to Study Objectives

In the 31 papers analysed (Table 7), we found that 83.87% (*n* = 26) had a main aim that was directly or indirectly related to park design.

Five papers (16.12%) were not related to park design. Instead, they focused on the observation of physical activity engagement in parks, seasonal park use, parental perceptions with regards to their children’s motor development and positive implications for obesity, park use from the perspective of the intergenerational relationships they cause, and the relationship between the number of parks and obesity.

### 3.3. Synthesis of the Reviewed Studies

As a synthesis of the data analysed in the previous section, we highlight that the variables most analysed in the 31 studies correspond to the park use group, shown in a total of 19 papers (61.3%): the characteristics of the park (*n* = 15; 48.4%), the perceptions of users, both children and adults (*n* = 9; 29%), the time of PA (*n* = 6; 19.4%) and the intensity of PA (*n* = 13; 41.9%). The data collection instruments for each block of variables were varied, with the following being predominant:

- Use of the park: Of the 19 papers that measured this variable, the most used instruments were the SOPARC (*n* = 11; 57.9%), interview (*n* = 5; 16.1%) and questionnaires (*n* = 4; 12.9%).
- Characteristics of the park: Of the 15 papers that analysed different characteristics, the most widely used instruments were questionnaires (*n* = 6; 40%) and SOPARC (*n* = 4; 26.7%).
- Perceptions of users, both children and adults: Out of nine papers, the most used instruments were interviews and questionnaires (*n* = 4; 44.4%), which were used in each of them.
- PA time: Of the six papers that analysed this variable, the most widely used instruments were the SOPARC (*n* = 3; 50%) and the MET-h (*n* = 2; 33.3%).
- PA intensity: Of the 13 papers that measured it, the most used instrument was the SOPARC (*n* = 11; 84.6%) and the SOPLAY and accelerometers, with (*n* = 3; 23.1%) each.

Of the sample object of this study (*n* = 31), 48.38% (*n* = 15) of the papers established a direct relationship between the design, characteristics and equipment of the park with an increase in visits, a greater and more effective use of the park, and a greater intensity of PA, with 32.25% (*n* = 10) being remodelled, innovative, contemporary and sustainable parks with special characteristics [4,53,62,64,69,72,75,77,80]. If the needs of users are also taken into account in the remodelling or design of new parks, 16.12% (*n* = 5) of the studies analysed concluded that it can create a positive effect on the number of visitors and, therefore, an increase in PA performed [49,66,71,83]. Regarding the characteristics that parks should have to encourage visits and their use, of *n* = 12 (38.7%) papers that addressed the age from three to six years, the main characteristics that were considered relevant in a park, according to the perceptions of the children’s companions, were: a safe environment (41.7%) that allows outdoor activity (25%), has green areas and forests (25%), close to their residence (25%), allows various activities (16.7%) and has swings (16.7%).

Of *n* = 9 (29%) papers that addressed the age of 6–12 years, the main characteristics that were considered relevant according to the perceptions of the children’s companions were: a safe environment (44.4%) with green areas and forests (44.4%) that have a variety of facilities and allow various activities (44.4%) and that have comfort zones (22.2%).

According to the perspective of the 6 to 12-year-old children themselves, in *n* = 2 (6.5%) papers found, we observed that their preferences were focused on large parks, selecting a variety of activities and having comfort zones.

From the point of view of the increase in visits and use of the park, according to observational studies of *n* = 12 (38.7%) papers that addressed the age of 3–6 years, the main characteristics that were considered relevant were: a variety of games and activities (33.3%), swings (33.3%), green areas (16.7%) and that they are well designed, even if the park is further away from their area residential (16.7%). However, of *n* = 9 (29%) papers covering the ages of 6 to 12 years, the observational studies highlighted important characteristics: the variety of play areas (55.6%), which allow for organised sports (44.4%) and that have green areas (22.2%).

Regarding the ability of parks to promote PA, of *n* = 7 (22.6%) studies whose objective was to achieve an increase in physical activity in children, 100% of them obtained positive results. In total, 85.7% of these studies were related to renovations, creation of innovative parks and special parks. Of *n* = 10 papers that measured the intensity of physical activity in children, 100% indicated an increase in moderate to vigorous intensity PA (MVPA); 66.7% of these studies referred to models or special parks.

### 3.4. Risk of Bias of the Studies

To establish the methodological quality of the study, the reliability of selection and detection was determined by two authors using Cohen’s Kappa statistical index (Kc). A value of Kc = 0.834 was obtained for the coding, which shows an acceptable concordance and was analysed using the statistical software IBM SPSS^®^ in its version 24.0 (IBM Corp., Armonk, NY, USA) for Windows.

## 4. Discussion

### 4.1. Evidence Summary

Public parks have been proposed as an option for health promotion when it comes to addressing the high rates of physical inactivity [85] in the general population, and especially in children [23] from the earliest ages. This is because parks are considered to offer safe spaces for exercise and are available to the entire population [45]. Therefore, they have the potential to restore the health and well-being of users, especially in large cities where the physical environment favours a sedentary lifestyle and associated diseases [86]. This systematic review aimed to contribute to the need to expand data on the characteristics that parks and their surroundings should have to facilitate and maximise the practice of PA in them. This need is reflected in previous reviews such as those by Zhang et al. [87], where the inconsistency presented by the findings on the environmental factors of the park and the neighborhood was highlighted. This review provides data focused on the needs of school-age children (preschool stage, from 0 to 6 years old, and primary stage, from 7 to 12 years old), although the data provided from the selection of studies does not allow for taking into account differences according to gender. The data obtained are not highly consistent due to the scarcity and variety of studies and the lack of specific data focused on these stages. On the other hand, the review provides data related to the interests of park users, both children and the adults in charge of running them, establishing differences according to these age ranges and providing data from opinion studies, as well as observational and experimental studies. Finally, the characteristics of the different park designs that favour the increase in PA, its intensity and the development of motor skills are established.

Among the findings, we highlight that, although the socioeconomic contexts analysed have been varied, it is observed that children living in low-income communities have a higher risk of obesity due to the associated health inequalities [88]. However, this is not supported by the low percentage of studies included in the review. These populations are served by fewer services, which also tend to be of lower quality [89]. Therefore, a park renovation can benefit the PA of children living in these settings. Focusing efforts on the remodelling and/or design of parks in areas of low socioeconomic status is essential from the perspective of health and PA. This is because improving public spaces in disadvantaged areas can increase recreational PA [4,62,64]. This is not only beneficial for children from disadvantaged backgrounds, but it also applies to other contexts where, as current data indicate, park use is higher among boys than girls [49,64,79]. Therefore, the consideration of user preferences can improve the use of parks regardless of gender, although this should be studied in a more in-depth way since the sample selected in this review is not sufficient.

Families and caregivers of children tend to agree on the positive contribution of public playgrounds to the development of social, motor, cognitive and physical skills [61]. Taking into account the opinions of local populations will allow for a better understanding and planning of park design, or if necessary, redesign. This will ensure optimal use at different ages and by all genders and will increase visits to the park, PA, development of motor skills, physical fitness and social interaction [57,62,81]. However, user opinions are often not taken into account, causing the parks to be underused [83] and attracting only a subset of neighborhood children [50].

The results of the analysed papers regarding to the perspectives of the users could be a starting point for new approaches directed to individual populations, depending on the social and economic context. However, less than half of the investigations considered included surveys or interviews to capture the opinions of users, whether they were adults (families or caregivers of children during leisure time) or the children themselves. In fact, only two studies specifically focused on the perceptions of children aged 7 to 12 years [57,68]. Qualitative research is important and provides a lot of data on park preferences from the users’ point of view. This would allow a more effective adaptation of the parks for the improvement of PA [49,51,57,66,71]. Thus, we consider it to be an essential aspect for future research.

The main data emerging from the research, after considering the users and their age, are the following:

According to the opinion of family members and caregivers of children from 0 to 6 years old, to encourage greater use of these spaces, playgrounds should offer opportunities for outdoor activities with green areas and forests [60,65,83], and be close to their residence or where their friends or relatives live (60,65,81], as this will allow them to interact with other children or with their parents. Parks should allow the performance of various activities and games that motivate children [60,69], with safety and cleanliness being another factor that encourages visits [65,83]. However, family members and caregivers aged 7 to 12 choose large parks (even if they are not located near their residential area) [60] and, with sports facilities and other amenities such as bathrooms, seats, fountains with drinking water, barbecues and gardens [49,57,78]. In addition, they must have areas that allow different age groups to exercise (multigenerational parks) [64], have different play areas [78], that are accessible [51,64,78], of high quality [83] and restrictions on dogs, vandalism, litter and fencing. This is in line with the review by Audrey and Batista-Ferrer [90] who stated that the provision of clean, safe and accessible public open spaces can offer opportunities for physical activity and social interaction, however, data obtained by Zhang et al. [87] did not find this to be a significant factor. Although most users of play areas prefer that the facilities are nearby, this is not a decisive characteristic when choosing a park [83], a fact that coincides with the findings obtained by Zhang et al. [87]. Parents’ preference for parks farthest from their residence, but that are better equipped, is because they think that these types of parks are more suitable for the whole family. This is because many children of this age tend to have siblings, so they seek more opportunities for activities than those offered by nearby but small parks [49].

Regarding the data obtained about the motivations of children from 0 to 6 years old to attend the parks, we did not find any study that examined their perceptions, which indicates the need for studies at these ages.

This may be due to difficulties in administering reliable measuring instruments at these ages. However, in the following range (8–12 years), although only two papers were found that analysed opinions at these ages, they showed a preference towards large parks with green areas or forests for playing, cycling or sports [57,83]. Parks should include play equipment that involves some risk and adventure, such as swings and large and tall slides [57] and that have other facilities (seating and picnic areas) that allow for social interactions and a place to go with friends, siblings or adult relatives [57,83]. Contrary to what is believed by parents and children of other ages [91,92], these children are not concerned about the lack of comfort, safety or cleanliness [57].

Regarding the characteristics obtained in the observational and experimental studies carried out in different types of parks (remodelled, innovative, special and traditional), this study provides, as a novelty, the characteristics that must be taken into account in the remodelling and innovation processes of traditional parks or in the creation of new parks that favour their use. Thus, the data reflect that large swings, 360-degree traditional swings, mazes, rocking chairs, sandboxes, adventure equipment (for example, climbing equipment) and games in nature should be incorporated [62,69], and fitness areas should be created [64]. Garden areas with spaces for community events [74] should be added, and shaded areas and different play spaces [80] should be created to facilitate unstructured play [81]. In addition, according to Roemmich and Johnson [56], ice rinks, hills for tobogganing, cross-country skiing and covered areas for physical activity should be provided and lighting improved to encourage active use of parks in colder areas during the winter.

These parks must also be safe to facilitate the practice of PA, especially when targeting women [51], and must have multigenerational accessibility [64]. The latter may be a key component for the more widespread adoption of healthy practices in urban parks. Differences are found between the different parks that children visit between the ages of 3 and 12. As children transition from preschool age (3–5 years) to elementary school age (6–9 and 9–11 years), they visit larger, better-equipped parks with sports fields and recreational activities that are more adventurous and challenging [57]. Comfort also becomes more important for this group, as reflected in the need for comfort facilities such as bathrooms, better lighting, etc. [60].

Regarding park designs that favour a greater commitment to more intense PA, the papers reviewed indicate a positive association between access to parks and PA in children and a lower obesity rate [61,68,69,71], although previous review studies [87] did not find accessibility a significant factor in park use for PA. The offer of services increases the use of parks and encourages PA [62,93], with this being especially important for girls [94,95]. The diversity of sports fields (basketball, handball, soccer, baseball, etc.) can increase PA [63,96], although other studies such as those carried out by Spengler et al. [81] and Floyd et al. [97] do not support this idea, finding that organised activity in parks is not related to an increase in PA in younger children. These children are hardly interested in structured PA, but instead prioritise free and spontaneous play. Issues such as security [98,99], poor maintenance of facilities [100,101] and lack of adequate supervision [72] have been identified as the main obstacles to the use of parks and, consequently, with a reduction in PA [63]. Thus, good security and access to playgrounds improve active play [74]. On the other hand, some characteristics of public parks (such as play areas) have been associated with higher energy expenditure compared to other characteristics [67]. In children under 13 years of age, traditional playgrounds with swings and climbing equipment favour PA [102].

The designed or renovated playgrounds (innovative parks, Troyan Park, pocket parks, playscape parks, Krajicek parks and renovated parks for cold areas) that meet the preferences of children clearly increase PA [4,53,56,62,64,69,77]. However, other studies did not find significant differences from traditional parks in relation to increased PA [95].

Regarding the characteristics of the park that can influence the levels and intensity of PA, the studies identified that swings and sports facilities, especially basketball courts, pedestrian paths and different types of terrain surfaces [63,67,74] (for example, grass, trees, hills, running water, and sand [103]) allow for a higher intensity of PA. The relationship between greenness and PA for children is similar to previous reviews, in which it is suggested that the green environment influences an increase in total PA [87]. Possibly the cause is the reduction of negative emotions and increased energy. Parks without benches [71,76] that include trails and cycling routes also increase the intensity of PA [75]. The relationship between trails/roads and park-based PA, also analysed by Zhang et al. [87] with consistent data, suggested that people are more likely to participate in walking, running and cycling in parks when trails/roads exist. Redevelopment of playgrounds is an effective strategy for increasing playground use and levels of moderate and vigorous PA in both boys [72] and girls [74]. However, according to the data of Reed et al. [79], the vast majority of observed PA is usually vigorous.

Finally, regarding motor skills development (MS), few studies have examined the role of playgrounds in the development of motor skills in children. Specifically, the present study has only identified two published works that detail the importance of playgrounds for this development [61,67]. It is necessary for parks to integrate a mix of structured and free play. At the same time, they must have equipment that allows directed and instructed play [67]. This is important because free activities, by themselves, do not promote the development of motor skills [104]. For this reason, contemporary playgrounds promote the development of locomotor skills such as walking and running, while adventure parks promote the development of skills such as climbing and maintaining balance [105]. The few papers found make these data not consistent, and more research is needed in the relationship of the characteristics of parks in relation to the development of motor skills.

When trying to establish concrete data on the perspectives and design of parks that promote children’s health, the main limitations encountered were related to the diverse nature of physical environments and socioeconomic contexts. Additionally, the target groups vary considerably in terms of age, race, and health behaviours. Research that takes gender into account is also lacking.

More studies are needed to examine specific age ranges in depth. Studies should take into account the preferences of both boys and girls and, more specifically, should measure levels of physical fitness and motor skills. These are good indicators because they are worked on during visits to the park and go beyond the simple measurement of users’ levels of physical activity.

### 4.2. Limitations

The limitations of the study are mainly centered on the lack of studies found, which also present very diverse data in terms of physical environments, target groups (sex, age and ethnicity) and health behaviours. These studies also present diverse research designs, making it difficult to obtain consistent data. There is a shortage of interventions that provide objective data on the effectiveness of park design in improving and increasing PA in school ages, so it has not been possible to carry out a meta-analysis.

## 5. Conclusions

Urban parks are a valuable resource for the promotion of PA in different socio-economic contexts and especially in the most disadvantaged populations. The construction or redevelopment of parks should consider the needs of the population and the socio-economic context. Involving community members in playground renovations can have a positive effect on park use and PA engagement in children.

Park selection differs depending on the age of users. Family members and caregivers of children aged 3–6 years prefer outdoor playgrounds with green areas and a variety of play areas. They prefer parks that are close to their homes that are safe. On the other hand, 8–12 year olds prefer larger parks with a variety of sports, comfort facilities and walking and cycling paths. It does not concern them if the park is far from their residence. Park safety is a highly valued aspect for parents of children in both age ranges.

Parks designed or remodelled to suit the preferences of children and their careers increase PA at different ages. This is, therefore, an effective strategy for maximising park use and PA engagement in children.

It is clear that aspects such as playground availability and proximity are associated with higher PA levels and, therefore, lower levels of childhood obesity. However, the gender of users was not a significant predictor of the PA levels observed in the parks included in the present study.

### Future Perspectives

Future studies should go deeper into the level of physical fitness and motor skills that are worked on during visits to the parks instead of measuring only the level of physical activity of users.

This systematic review is the prelude to a future project which will seek to analyse the reality of playgrounds in the city of Granada, as well as to plan interventions to optimise and promote the use and practice of PA in school-age children.

## Figures and Tables

**Table 1:** Data related to the selected studies.

Papers	Authors	Year	Country	Age	Sample *	Park Type **	Context ***
1	Flowers et al. [49]	2020	Australia	3–11	86 Ch (47 Ml; 39 Fm) 571 Pa	UP	H-M-L
2	Veitch et al. [57]	2020	Australia	8–12	30 Ch (53% Fm) 9 Pk	UP	H-M-L
3	Flowers et al. [60]	2019	Australia	3–11	375 Pa 317 Pk	UP	H-M-L
4	Gil- Madrona et al. [61]	2019	Spain	24–40	1.029 Ad 41 Pk	UP	H-M-L
5	Lal et al. [62]	2019	Australia	-	2 Pk	TUP vs. RUP	L
6	Marquet et al. [63]	2019	USA	5–10	16.577 Ch 120 Pk	TUP	L
7	Parra et al. [64]	2019	USA	-	599 Ad; 246 Ch 1 Pk	TP (RUP)	M
8	Quiao [65]	2019	China	3–9; 24–40	1.320 (1030 Ad; 290 Ch)	PR	H-M-L
9	Talarowski et al. [53]	2019	London and USA	-	16 Pk	TUP vs. RUP	H-M-L
10	Washington et al. [66]	2019	Australia	18–60+	386 U (61% Fm) 12 Pk	TUP	-
11	Adams, Veitch & Barnett [67]	2018	Australia	5–10	57(28 Fm; 29 Ml) 3 Pk	TUP-RUP-AP	-
12	Rossi et al. [68]	2018	Brazil	7–14	2.152 (56.5% Fm; 43.5% Ml)	PP/PR	H-M-L
13	Veitch et al. [69]	2018	Australia	1–12	2374 (49.6% Fm; 50.4% Ml) 2 Pk	TUP vs. RUP	-
14	Bezold et al. [70]	2017	USA	11–14	94.997 Ch	PP/PR	H-M-L
15	Kaymaz, Oguz, & Cengiz-Hergul [47]	2017	Turkey	6–12	418 Ch; 383 Pa 8 Pk	UP	M-H
16	McCarthy, Hughey & Kaczynski [71]	2017	USA	8–11	13.469 Ch 95 Pk	PR	L
17	Ou, J.Y. et al. [51]	2016	USA	≥18	354 U	UP	M-L
18	Boonzajer et al. [4]	2016	The Netherlands	0–15	20 Pk	RKF	L
19	Slater et al. [72]	2016	USA	U	78	PP/PR	M-L
20	Arroyo-Johnson et al. [73]	2016	USA	U	100 Pk	PP/PR	L
21	Baek et al. [74]	2015	USA	8–17	94 Ch 1 Pk	RUP	M-L
22	Schipperijn et al. [75]	2015	Denmark	U	3 Pk	BP	M-H
23	Roemmich & Johnson [56]	2014	USA	0–5; 6–12; 13–18; ≥19	16 Pk	PP	M-H
24	Roemmich, Beeler & Johnson [76]	2014	USA	0–12; 19+	1 Pk	PP/PR	M-H
25	Cohen et al. [77]	2014	USA	-	U 15 Pk	PKP	L
26	Nasar & Holloman [78]	2013	USA	9–10	304 Ch (14 Fm; 17 Ml); 75 Pa 14 Pk	PP/PR	L
27	Reed & Hooker [79]	2012	USA	6–12;13–20	(1668 Ml; 1184 Fm) 45 Pk	PP	H-M-L
28	Colabianchi et al. [80]	2011	USA	-	20 Pk	TUP vs. RUP	L
29	Spengler et al. [81]	2011	USA	0–10	3410 Ch 28 Pk	UP	L -H
30	Quigg et al. [82]	2010	New Zealand	5–10	184 Ch	PP/PR	L
31	Jansson & Persson [83]	2010	Sweden	3–6; 6–11	141 Ch 2 Pk	PR	M-H

Note: * children (Ch); male (Ml); female (Fm); parents (Pa); parks (Pk); adults (Ad); users (U); ** urban Park (UP); traditional urban park (TUP); remodelled urban park (RUP); Trojan Park (multigenerational) (TP); recreational park/playground (PR); adventure park (AP); public park (PP); Krajicek playgrounds—sustainable parks (RKF); bike park (BP); pocket park (PKP); *** low level (L); medium level (M); high level (H).

**Table 2:** Data related to the selected studies.

No.	Study Objective	St *	Variables	Instruments **	Conclusions
1	To identify the type of children who visit parks far from their homes and compare them with those who do not visit.	QT	Park size; distance; access (transport); areas for activity; quality and safety	Interviews; ad-hoc questionnaires; Google maps; VPA	Children and families are willing to travel further to visit larger parks with a variety of facilities and services (sports courts, bathrooms, water fountains, barbecues, picnic shelters...).
2	To identify the opinions of children from different social classes regarding the characteristics that influence their visits to the park, PA engagement in the park and social interaction.	QL	Visit frequency; time of PA; social interaction	Interviews; ad-hoc questionnaires	Children are attracted to parks that facilitate play, have elements of risk/adventure and are large enough to allow for a variety of physical and social activities.
3	To examine the characteristics of parks visited by children at different stages (3–5/6–8/9–11 years).	QT	Access (transport); areas for activity; quality and safety	Survey; ad-hoc questionnaires; Google maps; VPA	When children reach primary school age, they usually visit parks that are farther away from home and are larger, with sports and active recreation facilities.
4	To examine parents’ perceptions of the contribution of public parks to children’s motor, social and creative development and to reducing childhood obesity.	QT	Social skills, motor skills and perceptual-motor skills; creativity; obesity; sex and age; educational level and responsibility	Validated questionnaire	Women between the ages of 30 and 49, with high levels of education and high levels of participation in their children’s education, had more positive perceptions of the impact of public parks on children’s motor, social and creative development and the reduction of obesity in children.
5	To evaluate the profitability of installing an active playground in a large park in a low socio-economic area.	QT	Time spent engaged in PA; age; activity type; PA level	SOPARC; MET	The remodelling of the park, designed specifically for children, was effective in increasing their PA.
6	To examine the association between the characteristics of the park and its use by 5–10 year olds of different ethnicities	QT	Park characteristics and the use of different areas; age; race; activity type; PA level	SOPARC; MET	Significant associations were found between park use, PA levels and park area characteristics.
7	To investigate park use, and satisfaction and perceptions of park users regarding park improvements.	QT	Park use and characteristics of different areas; age; activity type; PA level	Intercept interviews; video; SOPARC	Multi-generational parks with access to various activities and fitness areas facilitate PA in different age groups and can provide social and physical health benefits.
8	To identify the preferences of parents of children (3–9 years) in terms of the availability, location, shape, characteristics, safety and comfort of parks.	QT	Location; design; operational characteristics; safety and comfort	Online survey	Preference is given to playgrounds that are close to home or playgrounds in central green residential areas which offer the possibility of outdoor activity, green areas and high levels of safety.
9	To compare and evaluate playground use and levels of moderate to vigorous PA in innovative versus traditional playgrounds.	QT	Park use; PA level	SOPARC; Video	The design of an innovative playground was associated with an increase in MVPA, however, playground size was more strongly associated with the number of visitors.
10	To learn about the use of public parks in residential areas and the social interactions that take place in this context.	QL	Use; park design; type and level of PA; social interaction	Ad-hoc questionnaire Intercept interviews	Public parks in residential neighborhoods were proven to be specific areas for PA and social engagement.
11	To examine whether the design of the park facilitated higher levels of PA and fundamental motor skill development.	QT	PA level; motor skills	GT1M; SOFIT	Park design contributes significantly to the daily PA needs of children but involves a limited number of motor skills.
12	To study the relationship between the use of public places for PA and active leisure with respect to distance and overweight/obesity indicators in school children from different areas.	QT	Distance; frequency of use; body mass index (BMI); waist circumference (WC)	Questionnaire	Living closer to parks/playgrounds was associated with lower BMI and waist circumference amongst school children from low-income families.
13	To evaluate the impact of a playground facility on park visits and physical activity engagement within the park.	QT	Frequency of use; PA levels	SOPARC; intercept interviews; objective monitoring	A well-designed playground facility can increase the number of visits to parks and PA engagement in children aged 1–12 years.
14	To assess whether increases in the density of parks, playgrounds and sports facilities in the surrounding areas of a school are related with improvements in school physical fitness.	QT	Density of parks, playgrounds and sports facilities; physical fitness levels	Built Environment & Health Research Group (Columbia Un) FITNESSGRAM Test	No clear patterns of association were observed between the density of recreational resources around the school and changes in the physical condition of students.
15	To investigate patterns and factors related with the green space use behaviours of children aged 6–12 years and their parents.	QT/QL	Parental leisure trends; green area use	Ad-hoc questionnaire; drawing and/or writing surveys; questionnaire	Children’s use of green spaces is strongly linked to the environmental attitudes of their parents and the characteristics of their physical environment.
16	To examine disparities in access and quality of play areas according to socio-demographic characteristics, and examine associations between access and quality of play areas and BMI.	QT	Access to playgrounds; playground quality; gender; race/ethnicity; socioeconomic status; BMI	GIS shapefiles; ArcGIS 10.2; CPAT	Children with lower quality play areas were more likely to be overweight than children without access to play areas. There were no significant outcomes regarding access/quality and weight as a function of economic status.
17	To investigate park use and PA engagement, and the relationship of this with exposure to community violence	QT	Age; sex; race/ethnicity; education; injury; employment status; seasonality	Questionnaire	Users prefer parks with a variety of facilities, green spaces and walking trails. Insecurity decreased all types of PA.
18	To examine whether park use and PA level in children is higher in Krajicek playgrounds compared with control playgrounds.	QT	Park use; PA level	Direct observation; SOPLAY	The study shows that children’s park use and PA level are higher in Krajicek playgrounds than in control playgrounds in disadvantaged neighbourhoods.
19	To compare park use and PA outcomes between a renovated playground that is adapted to the community and non-renovated parks.	QT	Park use and PA level; neighbourhood safety; distance to the park and climate	SOPARC	Involving the community in playground renovations can have a positive effect on park use and the intensity of the PA engaged in.
20	To assess the impact of playground safety and distance as part of the built environment on increased youth PA.	QT	Safety; maintenance; distance	Surveys; Google Earth	Disparities in playground safety and proximity reveal an opportunity to develop community-wide playground interventions for PA in youth.
21	To examine whether the particular design characteristics of parks facilitate greater PA intensity amongst young people.	QT	Park characteristics; PA level; MET minutes; age; sex	GPS receivers; accelerometers; GIS database	Park characteristics such as the complexity of surfaces, proximity to sports areas, playgrounds and paths allow for higher levels of PA.
22	To evaluate park use and PA levels of users of three new bicycle parks.	QT/QL	Park use; PA levels; time zone	SOPARC; on-site interviews	Park use and active participation of children and adolescents increased, particularly amongst boys; 63% of users were active during use.
23	To determine seasonal variation in park visits, the choice of services and PA intensity.	QT	Park use; age; sex, PA levels; heat sensation	SOPARC	Less total PA was engaged in during winter than during other seasons.
24	To examine the effects of an intervention based on the removal of seating on the time spent using the park and PA improvement.	QT	Park use; age; sex; PA levels	SOPARC	Adults were more physically active. Curtailing of the time made available to children to use and play in the park was reduced.
25	To assess the use of new pocket parks in low-income neighbourhoods.	QT	Age; sex; race/ethnicity; PA levels; park characteristics.	SOPARC; surveys	Pocket parks are perceived to be attractive and safe destinations and may increase PA by encouraging families to walk there with their children.
26	To uncover the main characteristics influencing the choice of playgrounds in African-American children and their parents.	QT	Park characteristics; park use	Survey; adapted SOPARC	Outcomes verified correlations between park suitability and provision of good quality and safe equipment.
27	To identify the most used areas of the park for PA and PA levels in children in 45 parks in a south-eastern community.	QT	Park use, Age; sex; race/ethnicity; PA level	SOPARC	The swing areas was the most used active setting by children, regardless of ethnicity.
28	To examine the influence of park or playground characteristics such as the amount, type and safety of equipment on park/playground use and PA level.	QT	Park characteristics; socioeconomic status; PA level	Environmental Assessment of Public Recreation Space Systematic Observation	The total number of features or amenities in the park/playground is associated with the use of renovated parks/playgrounds but not with PA levels.
29	To examine the PA levels in children in neighbourhood parks.	QT	Sex; race/ethnicity; neighbourhood income level; amount of shade; time of day; organised activity; park activity areas	SOPLAY;	Children’s PA engagement in parks in high-income neighbourhoods was higher than that of those in lower income areas. To increase PA, parks should provide courts, playgrounds and free play areas.
30	To identify the proportion of children’s PA that occurs in public parks with play areas.	QT	PA level; height; weight	Actigraph GT1M; Globalsat DG-100	A low proportion of children’s PA activity engagement occurs in parks with play areas (only 2% of total daily PA).
31	To assess whether the existing offer of traditional parks is adapted to the needs and preferences of different users.	QL/QT	Park characteristics; use and preferences; play areas and equipment	Questionnaire; interview; observation; GIS mapping technique	Users have different needs and preferences as a function of their age. The planning and management of playgrounds should take greater consideration of the needs of users and the local context.

Note: * Study type (ST); quantitative (QT); qualitative (QL); ** Victorian Planning Authority (VPA); System for Observing Play and Recreation in Communities (SOPARC); metabolic equivalents (MET); GT1M ActiGraph accelerometers; System for Observing Fitness Instruction Time (SOFIT); System for Observing Play and Leisure Activity in Youth (SOPLAY); Play and Leisure Observation System (ArcGIS); Community Park Audit Tool (CPAT).

**Table 3:** Types of parks analysed.

Park Type			Number of Papers	Percentage
Parks (urban, traditional)			10	32.25%
Recreational parks (playground)			11	35.48%
Other	Remodelled	2	10	32.25%
	Innovative	2		
	Sustainable park	1		
	Contemporary	1		
	Special: Bike park, pocket park, adventure park, Trojan Park	4		

**Table 4:** Socioeconomic context.

Socioeconomic Context:			Number of Papers	Percentage
All: Low, medium, high			9	29.03%
High	Medium-high	5	6	19.35%
	Medium	1		
Low	Medium-low	2	12	38.70%
	Low	10		
Unspecified			4	12.90%

**Table 5:** Variables examined in the considered research articles.

Variables		Number of Papers to Include This Variable	Percentage
Pertaining to features of the park	Features/equipment/quality/design	14	45.16%
	Security	6	19.35%
	Access/location/distance	8	25.80%
Pertaining to park use	Frequency/number/density	15	48.38%
	Activity zones	5	16.12%
	Time of day	2	6.45%
	Weather	2	6.45%
Pertaining to PA	PA time	1	3.22%
	PA type	8	25.80%
	PA intensity/PA level	17	54.83%
	Motor skills	2	6.45%
Pertaining to users	Age/gender	13	41.93%
	Ethnicity	7	22.58%
	Obesity/height	4	12.90%
	Social interaction	3	9.67%
	Socioeconomic status	5	16.12%

**Table 6:** Frequency of instrument use.

Instrument	Number of Papers	Percentage
Surveys	6	19.35%
Questionnaires (ad hoc and validated)	9	29.03%
Interviews	8	25.80%
Bespoke pen and paper audit tool	1	3.22%
Environmental Assessment of Public Recreation Space Systematic Observation (EAPRS)	1	3.22%
Field observation	2	6.45%
SOPARC	12	38.70%
SOFIT	1	3.22%
SOPLAY	2	6.45%
GOOGLE MAP	2	6.45%
Mapping GSI	1	3.22%
GOOGLE EARTH	1	3.22%
ArcGIS 10.2; 9.0	2	6.45%
RECEPTOR GPS (DNR Garmin Foretrex 201)	1	3.22%
Global Positioning System: Globals DG-100	1	3.22%
TRAIL MONITORS	1	3.22%
MET	2	6.45%
ActiGraph accelerometers GT1M	3	9.67%
TEST FITNESSGRAM	1	3.22%

**Table 7:** Research objectives.

Study Categories	Number of Papers	Percentage	Objectives	Number of Papers
Related to DESIGN	26	83.87%	Park characteristics/park design and use/PA increases	12
			Comparison: traditional vs. renovated parks	2
			Remodelling/intervention effects	5
			Impact of special parks	2
			Distance and park use Access and obesity	3
			Security and access PA level and violence	2
OTHER not related to design	5	16.12%	PA engagement in parks	1
			Park use as a function of seasonal effects	1
			Parental perceptions regarding their children’s motor development and the reduction of obesity	1
			The construction of intergenerational relationships as a result of park use	1
			The relationship between the number of parks and obesity	1

## Data Availability

Data is contained within the article or supplementary material.
